# Global Agricultural Land Resources – A High Resolution Suitability Evaluation and Its Perspectives until 2100 under Climate Change Conditions

**DOI:** 10.1371/journal.pone.0107522

**Published:** 2014-09-17

**Authors:** Florian Zabel, Birgitta Putzenlechner, Wolfram Mauser

**Affiliations:** Department of Geography, Ludwig Maximilians University, Munich, Germany; Potsdam Institute for Climate Impact Research, Germany

## Abstract

Changing natural conditions determine the land's suitability for agriculture. The growing demand for food, feed, fiber and bioenergy increases pressure on land and causes trade-offs between different uses of land and ecosystem services. Accordingly, an inventory is required on the changing potentially suitable areas for agriculture under changing climate conditions. We applied a fuzzy logic approach to compute global agricultural suitability to grow the 16 most important food and energy crops according to the climatic, soil and topographic conditions at a spatial resolution of 30 arc seconds. We present our results for current climate conditions (1981–2010), considering today's irrigated areas and separately investigate the suitability of densely forested as well as protected areas, in order to investigate their potentials for agriculture. The impact of climate change under SRES A1B conditions, as simulated by the global climate model ECHAM5, on agricultural suitability is shown by comparing the time-period 2071–2100 with 1981–2010. Our results show that climate change will expand suitable cropland by additionally 5.6 million km^2^, particularly in the Northern high latitudes (mainly in Canada, China and Russia). Most sensitive regions with decreasing suitability are found in the Global South, mainly in tropical regions, where also the suitability for multiple cropping decreases.

## Introduction

Natural constraints are limiting the land's suitability for agriculture and cultivation practices. They consist of prevailing local climatic, soil and topographic conditions determining the available energy, water and nutrient supply for agricultural crops. Besides natural conditions, complex interactions of social, economic, political, and cultural aspects determine whether and how land is used for agriculture. Agricultural land has become one of the largest terrestrial biomes on the planet, occupying approx. 40% of the land surface [1]. Thereby, a variety of different land use types and intensities determine heterogeneously distributed patterns, including e.g. the choice of crop varieties, irrigation practices, fertilization, terracing and the level of technological input [2]. Thus, natural constraints are to a limited extent suspended by human actions [3].

The demand for agricultural products is expected to increase by 70–110% by 2050, driven by a projected world population of 9 billion people, increasing meat consumption and a growing use for bio-based materials and biofuel [4]–[15].

An increase in agricultural production can be accomplished by agricultural intensification and expansion, while considering social and environmental externalities and changing climate conditions [5], [16]. Bruinsma [16] concluded that additionally 1.2 million km^2^ of converted land are projected to be necessary until 2030 and another 5% up to 2050 with most land expected to be transformed in South America and Sub Saharan Africa, while latest studies project an increase of cropland between 10-25% by 2050 compared to 2005 for different socio-economic and climate scenarios [17]. Nonetheless, the expansion of agricultural land into forested or protected areas must be viewed critically, in order to conserve valuable ecosystem services e.g. for regulating climate or conserving biodiversity [5]–[8].

Changing patterns of temperature and precipitation and man-made degradation affect the suitability of land for agricultural use. For example, 19-23 ha of suitable land are lost per minute due to soil erosion and desertification [18], [19]. Additionally, the area of suitable land is decreasing due to urbanization, with an estimate of 1.5 million km^2^ until 2030 [20], [21].

When focusing on the natural potentials of land for agricultural use, suitability analyses give local evidence on todays and future availability and quality. Thus, they help answering questions for managing a transition towards a more environmentally efficient and sustainable land use and involve better information on the global scale impacts of land use decisions [1].

The relationship between climate, soil, topography and agricultural suitability has long been recognized. As such, suitability analysis combine heterogeneous soil, terrain and climate information and determine whether specific crop requirements are fulfilled under the given local conditions and assumptions. A variety of regional suitability studies for specific crops exist [22]–[28], while only a few exist on a global scale and for a broad variety of crops [3], [29]–[31].

In the meantime, global soil and topography data are available at high spatial resolution and global climate models have improved their capabilities and spatial resolution. Previous analysis showed that questions of scale play a major role in suitability analysis as coarse data affect the validity of results [32]. In this context, we present our results in modelling global crop-suitability using a fuzzy logic approach at a spatial resolution of 30 arc seconds. The results of this approach include the potentially suitable area for agriculture differentiated for 16 crops for rainfed and irrigated conditions, the start of the growing cycles and the number of crop cycles. We analyze global distribution of agricultural suitability and changes until 2100 considering the numbers of crop cycles.

Thereby, we identify changes, opportunities and challenges in global agriculture related to the expansion of agricultural land competing with protected and forested areas as ecosystem services.

## Material and Methods

Local climate, soil and topography determine the natural suitability of land for agricultural use. Thereby, the climatic, soil and topographic requirements may vary over a wide range of different agricultural crops. This analysis investigates the suitability for the following 16 crops that are most important for the global economy, food security and biofuel issues (see Table 1).

**Table 1:** List of investigated food, feed and energy crops.

Crop name
Barley (*hordeum vulgare*)
Cassava (*manihot esculenta*)
Groundnut (*arachis hypogaea*)
Maize (*zea mays*)
Millet (*pennisetum americanum*)
Oil palm (*elaeis guineensis*)
Potato (*solanum tuberosum*)
Rapeseed (*brassica napus*)
Paddy rice (*oryza sativa*)
Rye (*secale cereale*)
Sorghum (*sorghum bicolor*)
Soy (*glycine maximum*)
Sugarcane (*saccharum officinarum*)
Sunflower (*helianthus annus*)
Summer wheat (*triticum aestivum*)
Winter wheat *(triticum gestivum)*

**Figure 1:**
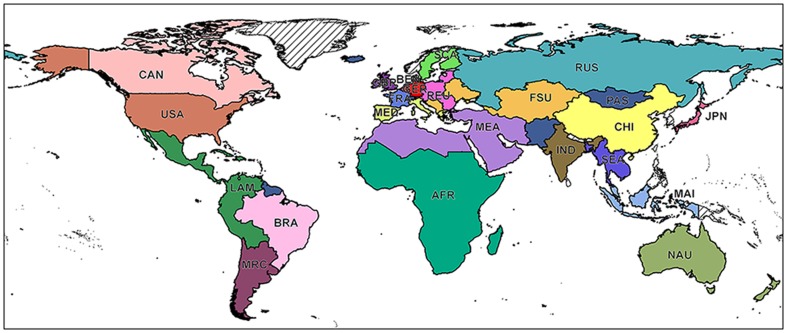
Map of the 23 world regions: AFR (Sub Saharan Africa), ANZ (Australia, New Zealand), BEN (Belgium, Netherlands, Luxemburg), BRA (Brazil), CAN (Canada), CHN (China), FRA (France), FSU (Rest of Former Soviet Union and Rest of Europe), GBR (Great Britain), GER (Germany), IND (India), JPN (Japan), LAM (Rest of Latin America), MAI (Malaysia, Indonesia), MEA (Middle East, North Africa), MED (Italy, Spain, Portugal, Greece, Malta, Cyprus), PAC (Paraguay, Argentina, Chile, Uruguay), ROW (Rest of the World), REU (Austria, Estonia, Latvia, Lithuania, Poland, Hungary, Slovakia, Slovenia, Czech Republic, Romania, Bulgaria), RUS (Russia), SCA (Finland, Denmark, Sweden), SEA (Cambodia, Laos, Thailand, Vietnam, Myanmar, Bangladesh), USA (United States of America).

**Figure 2:**
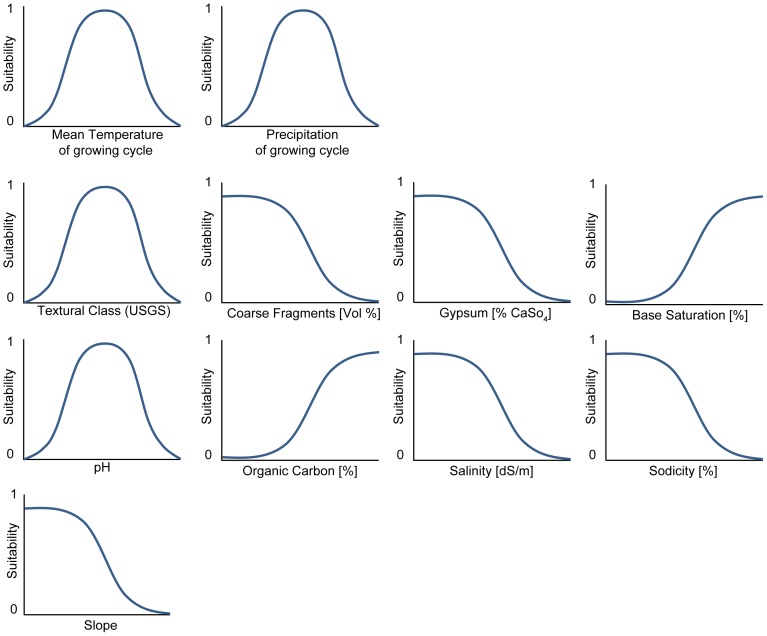
Membership functions for climatic, soil and topographic conditions.

We aggregated the world into 23 regions in order to regionally analyse the results (see Fig. 1). We applied a fuzzy-logic approach [33], [34] in order to calculate the crops' suitability on the globe at a spatial resolution of 30 arc seconds (0.00833°, approx. 1 km^2^ at the equator). The length of the growing cycle (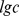) and the ‘*membership functions*’ that describe the crop-specific requirements for each of the crops during the growing period (Fig. 2) are derived from [35].

The membership functions representing climate constraints describe the degree of membership of each selected crop with regard to mean temperature and total precipitation during its respective growing cycle. Depending on the crop, membership functions have different curves according to [35]. Three shapes are in principle possible: ‘more is better’, ‘less is better’ and ‘optimum’. For temperature e.g., the suitability is increasing from a minimum towards an optimal temperature and again decreasing until a maximum temperature is reached (Fig. 2). Eight soil parameters are considered: texture, proportion of coarse fragments and gypsum, base saturation, pH content, organic carbon content, salinity, and sodicity. Terrain is considered by the slope. The fuzzy-logic approach calculates *fuzzy values* based on the ecological rules (between 0 and 1), which determine the crops' suitability on a specific location by the lowest membership value of all parameters.

An overview of the applied global datasets is given in Table 2. The climate data applied in this study are outputs from the global circulation model ECHAM5 of the Max-Planck Institute for Meteorology (MPI-M) [36], [37]. It uses radiative forcing, sea surface temperature and sea ice concentrations from a 20th century/SRES A1B scenario simulation. The 6-hourly dataset (temperature, precipitation) are converted to daily values for the climate period of 1981–2010 and 2071–2100. The daily data is spatially downscaled from its original resolution of 0.56° to 0.00833° (30 arc seconds), based on an approach by [38], using sub-grid terrain information provided by the SRTM-dataset [39]. A bias-correction is executed during the downscaling procedure for temperature and precipitation based on monthly derived factors from the WorldClim dataset [40].

**Table 2:** Applied global datasets.

Parameter	Source	Detailed Description
Climate	ECHAM5	[37]
Soil	Harmonized World Soil Database (HWSD)	[41]
Topography	Space Shuttle Topography Mission (SRTM)	[39]
Crop-requirements	FAO Land Evaluation Part III: Crop Requirements	[35]
Irrigation	Global Map of Irrigation Areas (GMIA) v5.0	[44]
Protected Areas	International Union for Conservation of Nature (IUCN) Protected Areas	[45]
Forested Areas	GlobCover 2009	[46]

Mean temperature (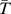) and total precipitation (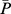) are calculated over the length of the growing cycle for each day of the year (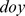) (see eq. 1 and eq. 2). Starting on the 1st of January (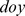 = 1), the growing cycle is shifted day by day until the 31st of December (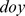 = 365). The suitability value (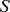) is calculated for each 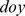 as in eq. 3 for 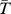 and 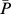 according to the membership function (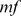). 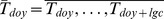
(eq.1)




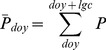
(eq.2)




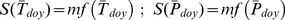
(eq.3)


Since the natural suitability of crop growth is limited by the minimum value, the smaller value of the temperature and precipitation fuzzy value determines the climate suitability 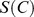 which is calculated for each 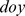 (eq. 4). 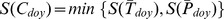
(eq.4)


Among all daily fuzzy values of 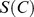 within the year, the maximum of 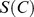 determines the climate suitability over the growing cycle and thus, the optimal start of the growing cycle (eq. 5) for cultivation of a single crop within the entire growing season. 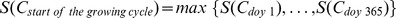
(eq.5)


In order to allow for the calculation of multiple cropping, the fuzzy values for each possible combination of days for the start of the growing cycle are tested as to how often they would fit within one year. The number of multiple cropping is selected that generates the highest accumulated value. Multiple cropping and the start of the growing cycle(s) are obtained for single, double and triple cropping. Hereby, the start of the growing cycle(s) in the context of this paper describes an optimal time for cultivation of a crop to reach the maximum suitability within a year. Crop mixing is not considered. Regarding temporal demands for technical field work, we assume a break of two weeks between crop cycles.

Moreover, the following assumptions are made: At least 20 mm of precipitation are required within the first two weeks of the growing season in order to provide enough soil moisture for germination. No day within the growing period must be below 5°C and below 1°C for winter crops. Vernalisation requirements are considered separately from the growing period for winter crops: Vernalisation period starts 150 days before the start of the growing period. At least 20 days below 5°C must exist during the vernalisation period and there must not exist more than 3 days below −30°C. In order to consider permafrost conditions that exclude agricultural use, mean annual temperature must not be below 0°C. Mean daily incoming solar radiation must exceed 60 W/m^2^ to provide enough energy for crop growth.

Thus, suitability values, number of crop cycles and the start of the growing cycle are calculated on each land surface pixel for both rainfed and irrigated conditions. For irrigated conditions, fuzzy values for precipitation are neglected during the calculation process. Due to a lack of global information on irrigation practices, we assume perennial irrigation on irrigated areas.

Besides climatic constraints, soil properties are limiting agricultural suitability. According to the membership functions (Fig. 2), the fuzzy values representing each of the soil properties are calculated. The minimum of the eight values represents the value of the soil suitability. Soil information was taken from the Harmonized World Soil Database (HWSD) [41], considering the topsoil (0–30 cm) of the dominant and all (up to 8) component soils at a spatial resolution of 30 arc seconds [42]. Within the calculation of soil suitability, fuzzy values of each of the component soils are calculated and weighted according to their share.

The suitability for crops to be cultivated is decreasing with increasing slope (see Fig. 2). The slope must not exceed 16% for the considered crops, except for oil palm and paddy rice. The slope was calculated and resampled to 30 arc seconds from Shuttle Radar Topography Mission (SRTM) data [39].

Across all climate, soil and topography fuzzy values, the lowest fuzzy value quantifies the crops' suitability at a certain location. The highest value across all crops determines the suitability for agriculture at a certain location.

This methodology does not allow for yield estimations, in which socio-economic and bio-physical aspects, which our approach does not consider, play an important role. However, this approach is well suited to draw conclusions about where areas are agriculturally suitable and how these areas may change with future climate conditions.

## Results

The Earth surface consists of 510 million km^2^ of which 149 million km^2^ are land surface. Up to 60°S, excluding Antarctica, and considering a lack of input data, in total 127.5 million km^2^ of land surface remain to be analyzed regarding their suitability for agriculture. We classified the results of the suitability analysis into four categories: not suitable (0), marginally suitable (>0.0), moderately suitable (>0.33) and highly suitable (>0.75).

### Comparison

Our results (further named GLUES in the Figures) highly correlate with existing studies, such as the GAEZ approach [29], when comparing the area of each of the four classified categories in each of the 23 World Regions (R^2^ = 0.99).

**Figure 3:**
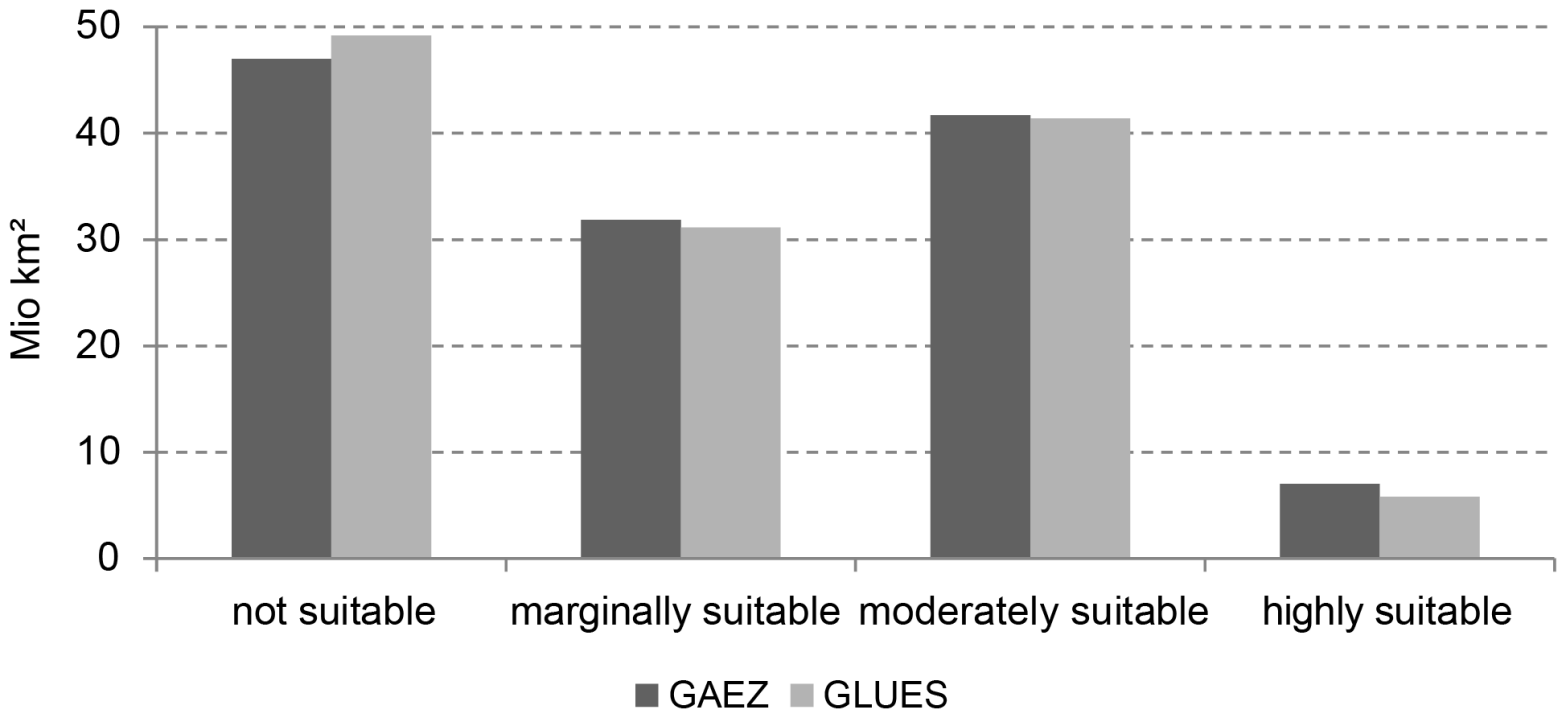
Global comparison of agriculturally suitable area between GAEZ (Baseline period 1961–1990) and GLUES (1961–1990).

**Figure 4:**
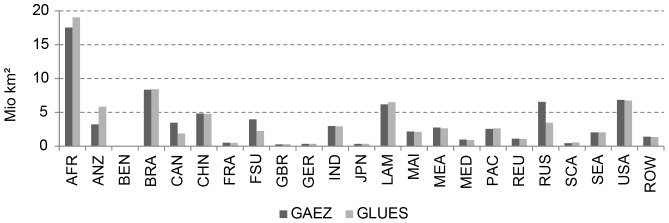
Comparison of total agriculturally suitable area of GAEZ (Baseline period 1961–1990) and GLUES (1961–1990) for different regions.

The global aggregation of the classified areas and the regional distribution of not suitable and suitable areas show a high level of agreement (Fig. 3 and 4). Compared to the distribution of global cropland in the year 2000 [43], our approach identifies 95.5% of current cropland as suitable.

### Rainfed

For the period 1981–2010, our suitability analysis shows that in total 77.7 million km^2^ are potentially suitable for purely rainfed agricultural cultivation, while 49.8 million km^2^ are not suitable for rainfed conditions (Table 3). Further, 30.6 million km^2^ are marginally suitable, 41.3 million km^2^ are moderately suitable and 5.8 million km^2^ are highly suitable (Table 3).

**Table 3:** Classified suitability considering rainfed conditions (1981–2010).

Not Suitable	Marginally Suitable	Moderately Suitable	Highly Suitable
49.8 million km^2^	30.6 million km^2^	41.3 million km^2^	5.8 million km^2^

### Irrigation

**Figure 5:**
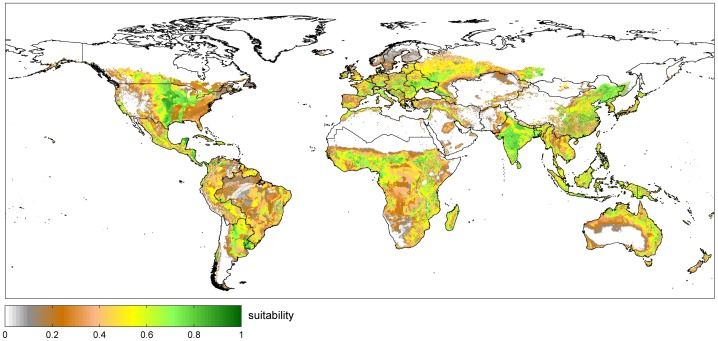
Agricultural suitability considering rainfed conditions and irrigated areas (1981–2010).

Irrigated agriculture produces 40% of the world's food (FAO) on 3.1 million km^2^ [44]. When considering irrigation, suitability is area weighted according to the fraction of rainfed and irrigated agricultural area (given by GMIA Version 5.0 [44]). Thereby, irrigation increases suitability on irrigated areas in global average by 0.13, adds 1.8 million km^2^ of suitable land (Table 4) and allows for multiple cropping on 1.2 million km^2^ (assuming sufficient water available for irrigation). Accordingly, huge areas e.g. in the Nile and Ganges delta are only becoming suitable due to irrigation. Overall, 79.6 million km^2^ are suitable with spatially varying patterns (Fig. 5).

**Table 4:** Classified suitability considering rainfed and irrigated conditions (1981–2010).

Not Suitable	Marginally Suitable	Moderately Suitable	Highly Suitable
48.0 million km^2^	31.8 million km^2^	41.8 million km^2^	5.9 million km^2^

Figure 5 represents the global distribution of agricultural suitability as a result of local climate, soil and terrain conditions. In boreal regions, the growing season over all stages of phenology usually is too short for cultivation. The temperate zones seasonally have adequate temperatures and enough precipitation and often sufficient soil, while in subtropical regions, the annual distribution of precipitation strongly determines crop growth and soils often are alkaline. In inner tropics have adequate temperature and moisture throughout the year, but soil quality often restricts cultivation due to low organic content and acidity [3].

### Protected Areas

Protected areas globally account for 8.3 million km^2^. Information on actual protected areas is gathered from IUCN [45]. When excluding protected areas from the suitability calculation, 74.8 million km^2^ remain suitable for cultivation. Thereby, protected areas are mainly situated in not suitable or marginally suitable areas (Table 5). Only 2% (0.2 million km^2^) of the global protected area are located on land highly suitable for agriculture, 25% (2.1 million km^2^) are on moderately suitable land, 30% (2.4 million km^2^) on marginally suitable land while 43% (3.6 million km^2^) are situated on unsuitable land. Overall, only 57% of global protected areas are suitable for agriculture.

**Table 5:** Classified suitability for 1981–2010 considering rainfed and irrigated conditions, excluding protected areas.

Not Suitable	Marginally Suitable	Moderately Suitable	Highly Suitable
44.4 million km^2^	29.4 million km^2^	39.7 million km^2^	5.7 million km^2^

### Forested Areas

Dense forests are highly important to provide numerous ecosystem services. Densely forested areas account for 23.3 million km^2^ according to GlobCover [46] and 23.5 million km^2^ according to [47]. GlobCover defines forests as being dense when 75% of the pixel is forest [46]. Only 1.5 million km^2^ or 6.2% of the global densely forested areas are currently protected.

4.9% (1.1 million km^2^) of the densely forested areas (excluding forests within protected areas) are located in highly suitable land, 49.4% (11.1 million km^2^) in moderately suitable land, 37.5% (8.4 million km^2^) in marginally suitable land and only 8.2% (1.9 million km^2^) are situated on unsuitable land. Overall, 92% of densely forested areas are potentially suitable for agriculture which indicates that global forests are subject to increasing societal stress.

### Current Use of Suitable Land and Trade-Offs

**Figure 6:**
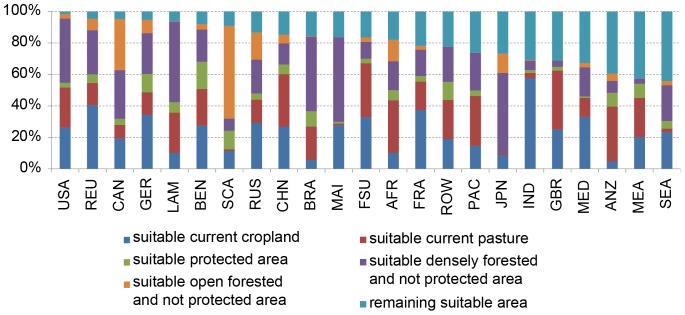
Current use of suitable areas (1981–2010), considering forest cover [46], protected areas [45] and current pasture and cropland (Ramankutty *et al.*, 2008). If forested areas are agriculturally suitable and protected, they are attributed to ‘suitable protected area’.

When excluding both, protected areas and dense forests from the suitability calculation, 54.1 million km^2^ remain suitable (Table 6). In comparison, currently used agricultural land (including pasture) today covers 49.1 million km^2^, of which 15.5 million km^2^ (status for 2011) are arable land (land under temporary and permanent crops; double-cropped areas are counted only once) [48]. Accordingly, 91% of all suitable land is already occupied by agriculture when today's protected and densely forested areas are preserved in the future. This illustrates that agricultural expansion is only possible by substituting other uses/covers of land which causes high social and ecological externalities. Figure 6 gives an overview of the current use/cover of suitable areas in the different regions of the world.

**Table 6:** Classified suitability for 1981–2010 considering rainfed and irrigated conditions, excluding protected and densely forested areas.

Not Suitable	Marginally Suitable	Moderately Suitable	Highly Suitable
42.6 million km^2^	21.0 million km^2^	28.6 million km^2^	4.6 million km^2^

Figure 6 shows, that the current fraction of suitable area, which is not protected or dense forest is highly variable across regions. The most efficient use of current agriculturally suitable land is obvious in the USA, where only 2% of currently suitable land is not yet used or protected/dense forest.

In Africa, about 20% of the agriculturally suitable area is currently not used for agriculture or is statistically not recorded in the data of currently used agricultural land (Ramankutty *et al.*, 2008). This shows the extraordinary potentials of Africa for future expansion of agricultural land. However, agricultural expansion would always take place at ecological costs (e.g. conversion of tropical rainforest, grassland and savannah). In Latin America large suitable areas are protected or covered with dense forest and the current fraction of remaining suitable area is smaller than in Africa, India is the prototype of a country, which is already using very large parts of its suitable agricultural land - and by for using the largest proportion (58%) of current cropland. Australia and larger parts of Asia still have reasonable land resources left for future expansion (Fig 6).

### Future Change

For the investigation of future agricultural suitability for the time-period 2071–2100 as determined by the simulated climate effects of the SRES A1B emission scenario, we assume no changes in irrigated areas, soil properties, terrain or any adaptations, such as crop breeding. As result, when again excluding protected and densely forested areas, the global area being highly suitable for agriculture decreases from 4.6 to 3.9 million km^2^, while marginally and moderately suitable areas increase (Table 7). In total, agriculturally suitable areas increase by 4.8 million km^2^ due to the selected climate change scenario. However, most of the additional area is only marginally suitable for agricultural use.

**Table 7:** Classified suitability for 2071–2100 considering rainfed and irrigated conditions, excluding protected and densely forested areas.

Not Suitable	Marginally Suitable	Moderately Suitable	Highly Suitable
37.8 million km^2^	24.8 million km^2^	30.2 million km^2^	3.9 million km^2^

**Figure 7:**
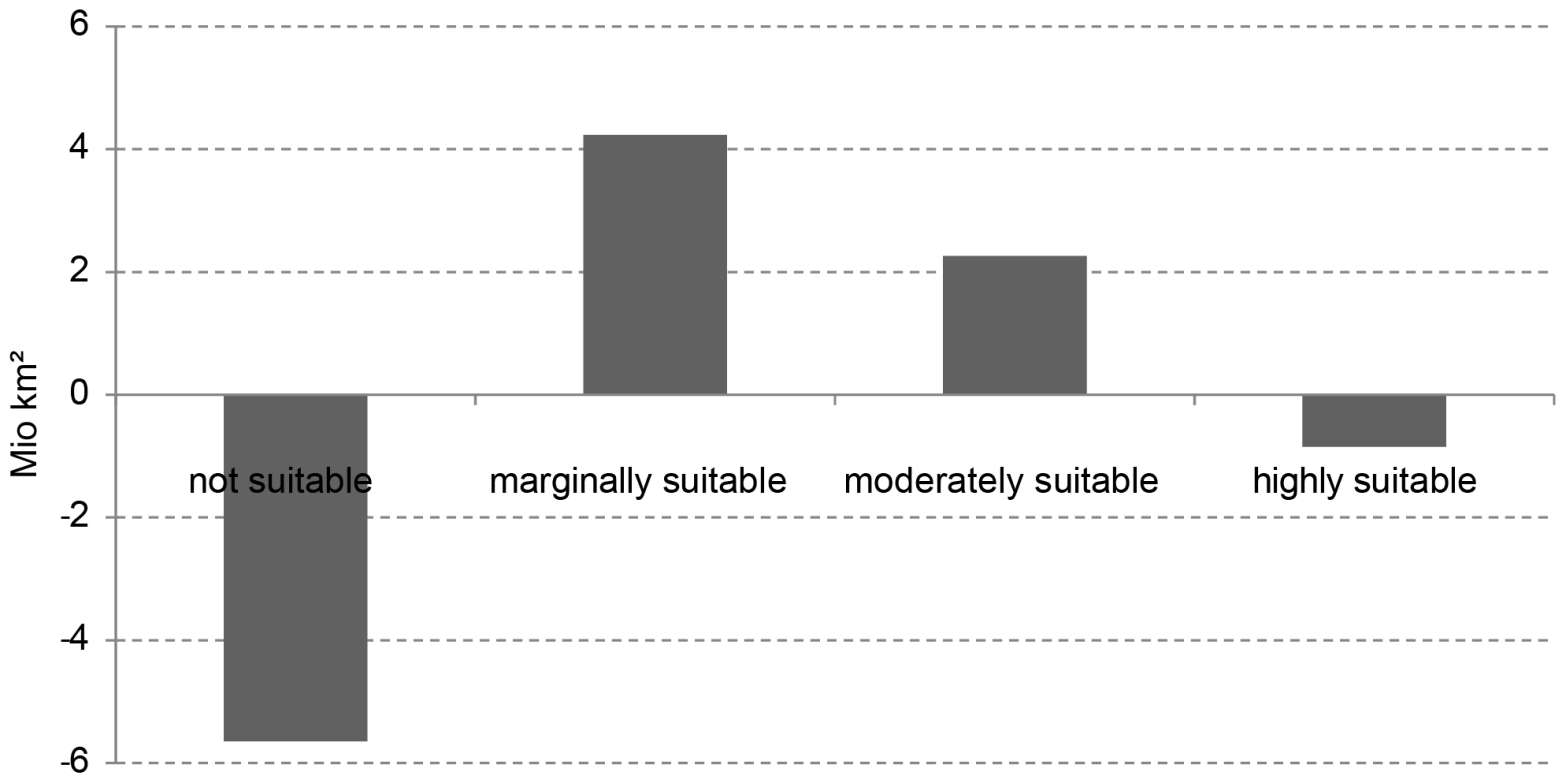
Global changes in agricultural suitability categories (million km^2^) between 1981–2010 and 2071–2100.

Without excluding any areas, the impact of climate change increases the potentially suitable areas on the globe by 5.6 million km^2^. Marginally suitable areas increase by 4.2 million km^2^, moderately suitable areas increase by 2.3 million km^2^, while highly suitable areas decrease by 0.8 million km^2^ (Fig. 7).

**Figure 8:**
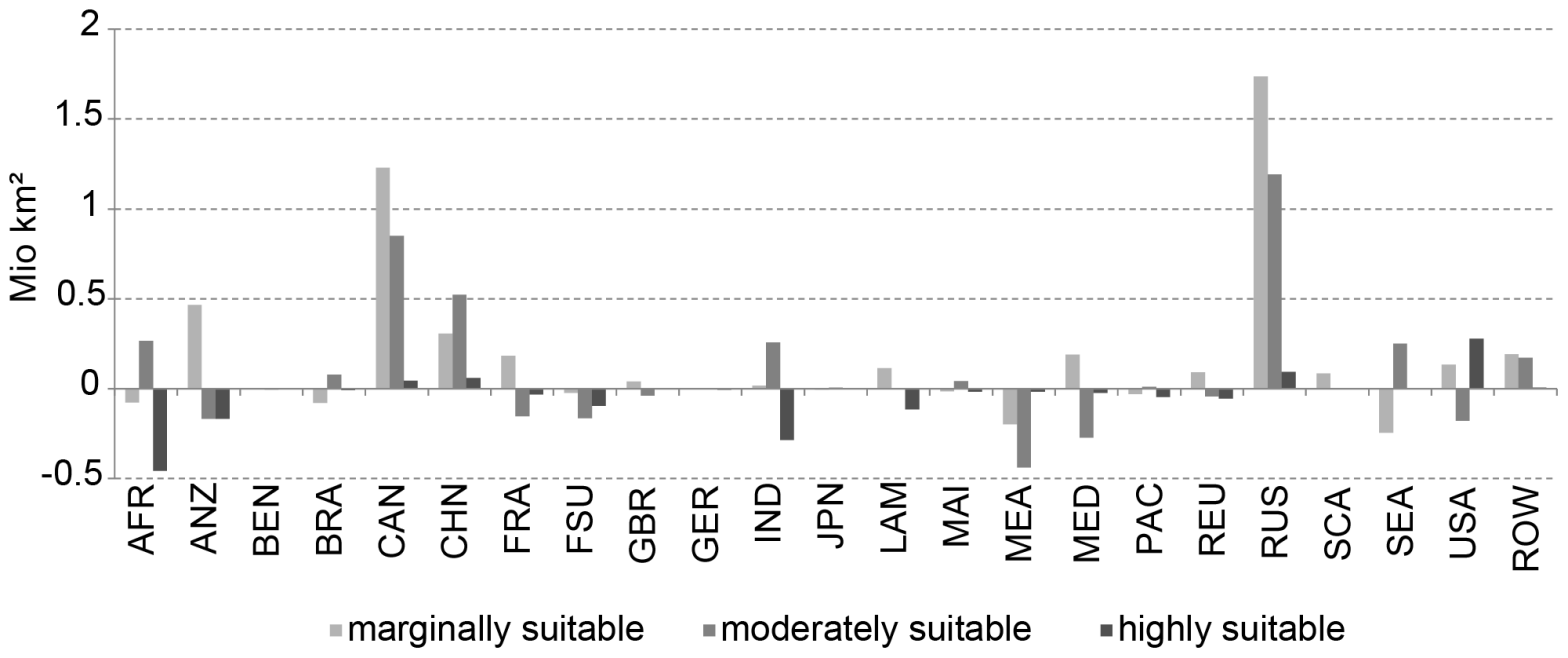
Regional change in agricultural suitability categories (million km^2^) between 1981–2010 and 2071–2100.

A more regional analysis shows that the world is divided into regions that receive additional suitable land and regions where land that used to be suitable turns into not suitable land (Fig. 8). Regions in the northern hemisphere, such as Canada (+2.1 million km^2^ of suitable land), Russia (+3.1 million km^2^) and China (+0.9 million km^2^), benefit most.

**Figure 9:**
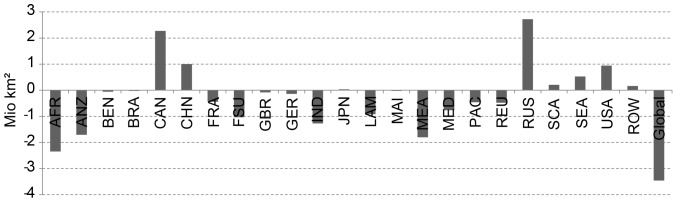
Regional change of agriculturally suitable area due to A1B climate change scenario between 1981–2010 and 2071–2100.

On the global scale, suitability improves on 18.7 million km^2^ and worsens on 22.2 million km^2^. In total, the area with decreasing suitability is 3.5 million km^2^ more than the area with increasing suitability (Fig. 9). The highest absolute net loss of suitable areas is found in Sub-Saharan Africa.

**Figure 10:**
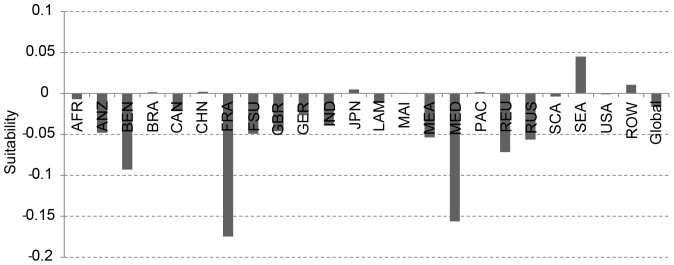
Regional changes in the average suitability between 1981–2010 and 2071–2100.

**Figure 11:**
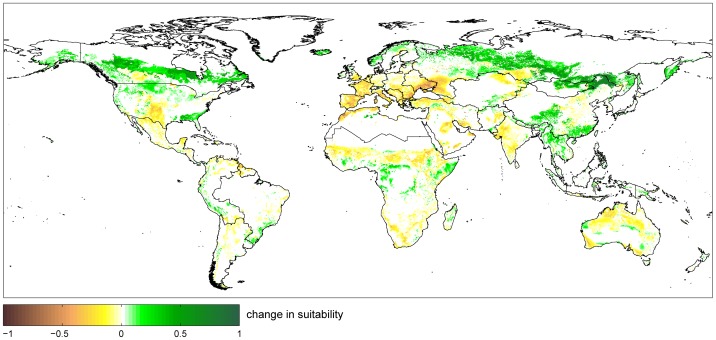
Change in agricultural suitability between 1981–2010 and 2071–2100. Green areas indicate an increase in suitability while brown areas show a decreasing suitability.

Thereby, the globally averaged suitability value (averaged over all suitable areas), decreases from 0.41 to 0.39. The greatest losses of suitability are simulated for France and the Mediterranean (Fig. 10). The changing suitability is mapped in Fig. 11.

### Growing Cycle and Multiple Cropping

**Figure 12:**
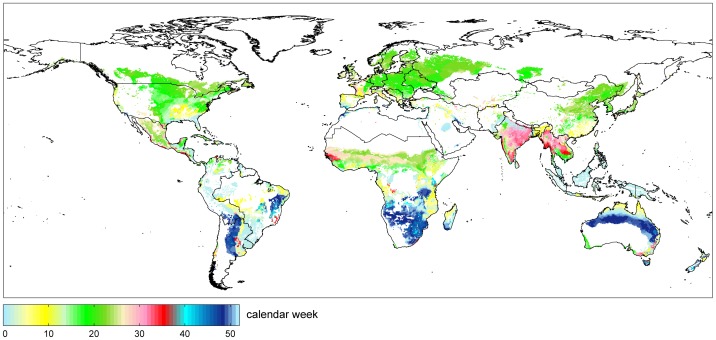
Start of the growing cycle for maize (1981–2010). The start of the growing cycle is illustrated for rainfed conditions and for irrigated conditions on predominantly irrigated areas (irrigated area > 50%). In case of multiple cropping, the map shows the start of the first growing cycle.

The seasonal development of temperature and precipitation determines the length of the growing season, the start of the growing cycle and the potential number of annual cropping. Thus, the option of multiple cropping represents an important measure for farmers to increase production. Figure 12 shows the spatial distribution of the start of the growing cycle for the time period 1981–2010, exemplarily for maize.

**Figure 13:**
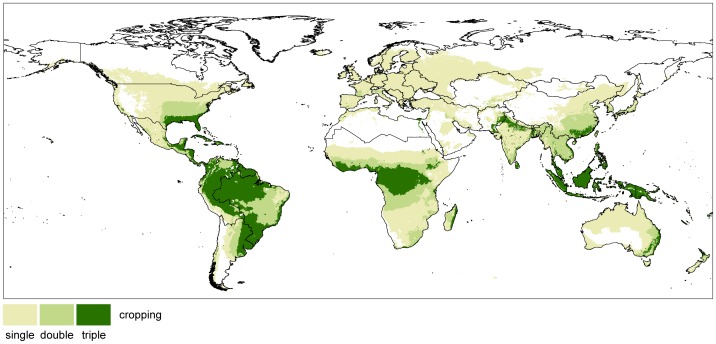
Suitable areas for single, double and triple cropping (1981–2010). Multiple cropping is illustrated for rainfed conditions and for irrigated conditions on predominantly irrigated areas (irrigated area > 50%).

Changing climate does not only affect the suitability of land, but also the start and length of the growing cycle. As an example, the start of the growing cycle for maize in Germany shifts in average 23 days earlier in time, when comparing the period of 2071–2100 with 1981–2010. The shift of growing cycles again influences the possibility for multiple cropping. Today's maximal achievable multiple cropping according to the course of temperature and precipitation is shown in Fig. 13.

Our results suggest that climate change has huge impacts on the areas suitable for multiple cropping under the assumed climate scenario. Until 2100, 6.0 million km^2^ are globally lost for triple cropping until 2100, while the area which is suitable for double cropping increases by 2.3 million km^2^. Multiplying the area with the number of cycles, this means a global decrease of 13.4 million km^2^. Most of the increase in double cropping areas results from the transformation from triple to double cropping. Again, no change in irrigation is assumed in this calculation.

**Figure 14:**
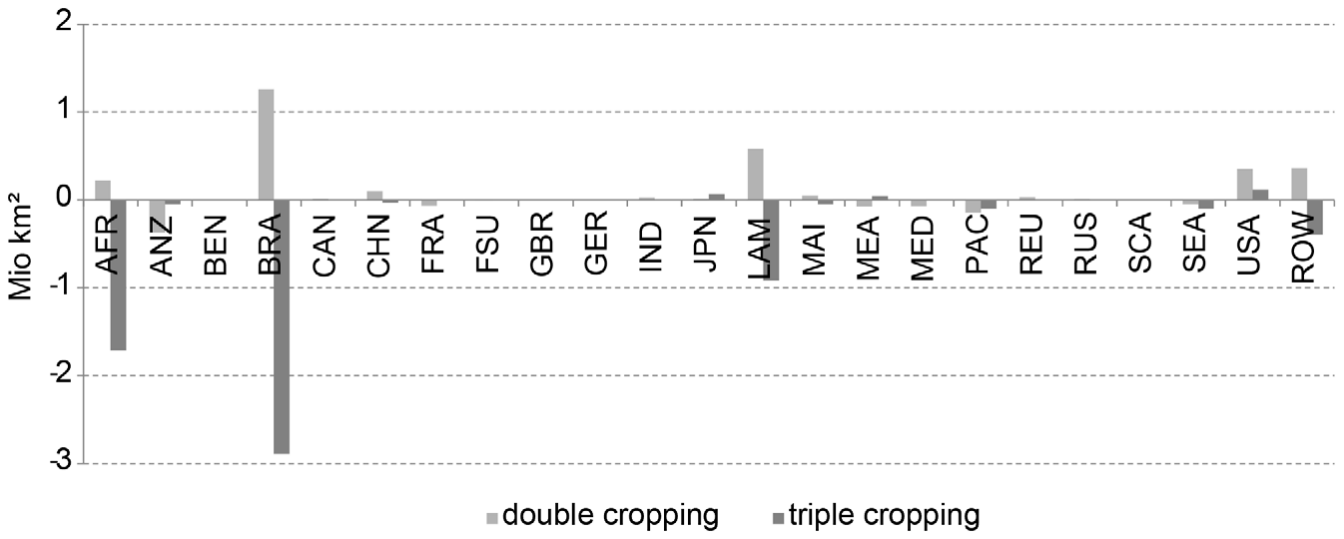
Change in area suitable for double and triple cropping (million km^2^) between 1981–2010 and 2071–2100.

The largest decrease in multiple copping area can be found in Brazil (BRA) and in Sub-Saharan Africa (AFR), where areas suitable for triple cropping decrease by 1.7 (AFR) and 2.9 million km^2^ (BRA) (Fig. 14), while the area for double cropping increases by 0.2 and 1.3 million km^2^, respectively. In total, this means a decrease of multiple cropping area by 1.5 (AFR) and 1.6 million km^2^ (BRA). This is equivalent to the amount of 4.7 and 6.1 million km^2^ respectively, which are lost for agriculture, when multiplying the area with the number of possible crop cycles. This corresponds to 20.2 (AFR) and 28.8% (BRA) of today's potentially suitable area for multiple cropping. In the same manner, France (FRA) and the Mediterranean (MED) lose 24.1 (FRA) and 13.2% (MED) of their total equivalent area when considering the change of multiple cropping, which means a decrease by 93 (FRA) and 55% (MED) according to the multiple cropping area of 1981–2010. Regions where areas that potentially allow for more than one crop cycle increase due to climate change are CHI, IND, JPN, MEA, REU, RUS and USA, while the total area considerably increases mainly in the USA for both, double (0.35 million km^2^) and triple (0.12 million km^2^) cropping (Fig. 14).

## Conclusions

The analyses of the present situation demonstrats that there is extraordinary potential e.g. for Sub Saharan Africa for future expansion of agricultural land without expanding into protected or forested areas. Further research is necessary to identify the environmental and social costs and consequences of agricultural expansion in these regions. Also further investigation is needed to give answers on how this land could be managed sustainable with benefit to local food systems and socio-economy.

Our results show at high spatial resolution how agricultural suitability may change until 2100 due to changing climate under the chosen scenario (SRES A1B), assuming no adaptation measurements by farmers. First, suitable areas increase especially in the northern regions such as Canada, China and Russia, where new land will be available for agricultural use. The increase in suitable areas mainly takes place in sparsely populated areas, which could imply a lack of labor for open up new agricultural land and prepare soils. Certainly, it will be related with high investment costs and it will take a long time to extend agriculture here. Secondly, global average suitability decreases under the chosen climate scenario. Especially the extend of highly suitable areas is reduced by the effect of climate change. Finally, suitable areas indirectly are reduced due to a substantial global reduction of the suitability for multiple cropping, especially in Sub Saharan Africa, and Brazil.

Overall, the Global North regionally increases suitability and the number of crop cycles, while the Global South and the Mediterranean area lose agriculturally suitable land without adaptations. This will decisively affect smallholder farmers as their options for adaptations through e.g. irrigation are limited.

Scientific knowledge on the geographical distribution has decisively being increased with the availability of global data sets, also based on remote sensing. The tensions between both limits of land expansion and intensification within the context of sustainable agricultural intensification stresses the ongoing debate on global land management, considering the complex interplay and trade-offs between different uses of land and ecosystem services.
