# Correction: Global Agricultural Land Resources – A High Resolution Suitability Evaluation and Its Perspectives until 2100 under Climate Change Conditions

**DOI:** 10.1371/journal.pone.0114980

**Published:** 2014-12-02

**Authors:** The PLOS ONE Staff

There are errors in Figure 1. The authors have provided a corrected version here.

**Figure 1:**
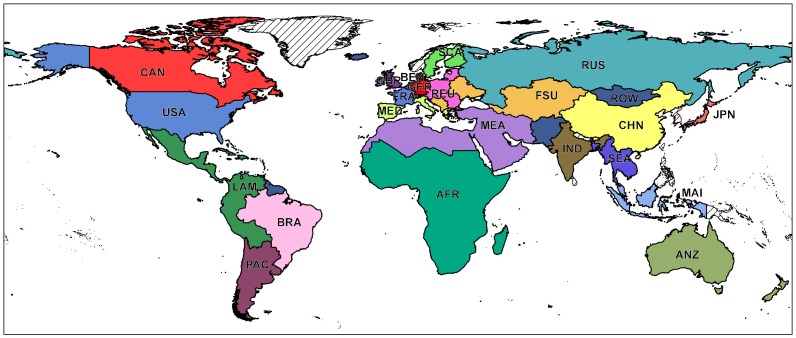
Map of the 23 world regions: AFR (Sub Saharan Africa), ANZ (Australia, New Zealand), BEN (Belgium, Netherlands, Luxemburg), BRA (Brazil), CAN (Canada), CHN (China), FRA (France), FSU (Rest of Former Soviet Union and Rest of Europe), GBR (Great Britain), GER (Germany), IND (India), JPN (Japan), LAM (Rest of Latin America), MAI (Malaysia, Indonesia), MEA (Middle East, North Africa), MED (Italy, Spain, Portugal, Greece, Malta, Cyprus), PAC (Paraguay, Argentina, Chile, Uruguay), ROW (Rest of the World), REU (Austria, Estonia, Latvia, Lithuania, Poland, Hungary, Slovakia, Slovenia, Czech Republic, Romania, Bulgaria), RUS (Russia), SCA (Finland, Denmark, Sweden), SEA (Cambodia, Laos, Thailand, Vietnam, Myanmar, Bangladesh), USA (United States of America).

The legend for Figure 9 is incorrect. The complete, correct Figure 9 legend is: Regional net balance between improving and degrading areas of agricultural suitability due to A1B climate change scenario conditions between 1981—2010 and 2071—2100.

**Figure 9:**
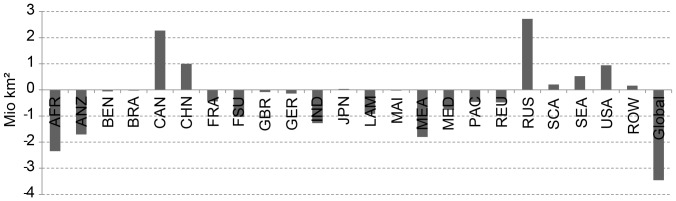
Regional net balance between improving and degrading areas of agricultural suitability due to A1B climate change scenario conditions between 1981—2010 and 2071—2100.
